# Acupuncture and Moxibustion for Poststroke Depression: Systematic Review

**DOI:** 10.2196/76577

**Published:** 2025-10-16

**Authors:** Lu Meng, Chuang-Long Xu, Xiao-Xu He, Xiao-Chan Tan

**Affiliations:** 1 Ningxia Hui Autonomous Region Hospital of Traditional Chinese Medicine, Ningxia Hui Autonomous Region Academy of Traditional Chinese Medicine, Yinchuan, China; 2 Hangzhou Red Cross Hospital (Zhejiang Hospital of Integrated Traditional and Western Medicine), Hangzhou, China; 3 The First Affiliated Hospital of Zhejiang Chinese Medical University (Zhejiang Provincial Hospital of Chinese Medicine), No. 54, Post Road, Shangcheng District, Zhejiang, Hangzhou, 310006, China, 86 18813080236

**Keywords:** acupuncture, humans, poststroke depression, systematic review, moxibustion

## Abstract

**Background:**

Poststroke depression (PSD) is a common complication following stroke. In recent years, several systematic reviews have evaluated the effects of moxibustion and acupuncture on PSD; however, their findings have been inconsistent.

**Objective:**

This overview of systematic reviews aimed to assess the methodological quality, reporting quality, and strength of evidence of existing systematic reviews on acupuncture and moxibustion for PSD. In addition, this study also analyzed the limitations of previous studies and suggested directions for future research.

**Methods:**

Systematic reviews concerning acupuncture and moxibustion for PSD published before August 10, 2024, were identified from 8 databases, including PubMed, Embase, Cochrane Library, Web of Science, China National Knowledge Infrastructure, Wanfang, VIP Database, and Chinese Biomedical Literature Database. Eligible studies included systematic reviews and meta-analyses of randomized controlled trials comparing moxibustion and acupuncture for the treatment of PSD. The methodological quality, reporting quality, and evidence quality were evaluated using AMSTAR 2 (Assessment of Multiple Systematic Reviews-2), PRISMA (Preferred Reporting Items for Systematic Reviews and Meta-Analyses) 2020, and GRADE (Grading of Recommendations Assessment, Development and Evaluation), respectively. The corrected covered area was calculated to assess overlap among the included primary studies.

**Results:**

A total of 24 studies were included. According to the Assessment of Multiple Systematic Reviews-2 assessment, all studies were rated as having “low” or “critically low” methodological quality. Based on PRISMA, 1 study demonstrated seriously inadequate reporting quality, while 21 studies had partially inadequate reporting quality. The quality of evidence in the included reviews ranged from very low to moderate. Most of the primary outcomes exhibited mild to moderate overlap among studies.

**Conclusions:**

Most of the included systematic reviews indicated that acupuncture may be beneficial for PSD. Nevertheless, the methodology, reporting, and evidence quality of these reviews require improvement. Stronger evidence will depend on the conduct of larger, multicenter, rigorously designed randomized controlled trials, as well as high-quality systematic reviews.

## Introduction

Poststroke depression (PSD) is a prevalent neuropsychiatric complication following stroke, characterized by depressed mood, loss of interest, and diminished quality of life during the recovery process [1]. PSD not only impairs patients’ quality of life but also exacerbates cognitive dysfunction, delays neurological recovery, and elevates the risk of recurrent stroke and mortality [2]. The pathogenesis of PSD remains incompletely understood but is thought to involve a combination of stroke-induced damage to emotional regulatory brain regions, resulting in neurotransmitter imbalances, reduced ability to perform daily functions, and insufficient social support [3].

Globally, the prevalence of PSD ranges from 17% to 34.3% [4]. Its incidence is influenced by factors such as age, gender, education level, marital and economic status, and stroke type [5]. The mainstays of Western medical management for PSD include pharmacotherapy, most notably selective serotonin reuptake inhibitors and norepinephrine-dopamine reuptake inhibitors, and psychotherapeutic approaches such as cognitive-behavioral and interpersonal therapies [6,7]. However, pharmacotherapy is frequently associated with adverse effects such as dry mouth, constipation, dizziness, somnolence, sexual dysfunction, and, in some patients, lack of efficacy or development of drug resistance [8-10].

Acupuncture, an integral part of traditional Chinese medicine, has demonstrated beneficial effects for PSD. Clinical evidence suggests that acupuncture can alleviate depressive symptoms, improve quality of life, and reduce the adverse effects induced by pharmacological agents [11,12]. The putative mechanisms include modulation of neurotransmitter systems, enhancement of cerebral blood flow, and regulation of immune function [13]. Numerous clinical trials and case studies point to acupuncture and moxibustion as effective interventions for PSD [14,15]. In recent years, multiple systematic reviews have synthesized the evidence on acupuncture and moxibustion for PSD, occasionally yielding inconsistent or conflicting results [16,17].

Therefore, this study presents an overview of systematic reviews that comprehensively summarizes the current state of research on acupuncture and moxibustion for PSD. By synthesizing existing evidence, it aims to provide a scientific foundation for clinical practice, clarify methodological limitations of previous studies, and propose recommendations for future research.

## Methods

### Protocol and Registration

The protocol for this overview of systematic reviews was registered with PROSPERO (International Prospective Register of Systematic Reviews; CRD42024576753). The reporting of this study adheres to PRISMA (Preferred Reporting Items for Systematic Reviews and Meta-Analyses) 2020 guidelines [18].

### Data Sources and Search Strategy

A total of 8 electronic databases were searched: PubMed, Embase, Cochrane Library, Web of Science, China National Knowledge Infrastructure, Wanfang, VIP Database, and Chinese Biomedical Literature Database. The search included records published up to August 10, 2024. Keywords used were acupuncture, moxibustion, electroacupuncture, manual acupuncture, acupoint catgut embedding, systematic review, meta-analysis, and PSD. Detailed search strategies for each database are provided in Table S1 in Multimedia Appendix 1.

### Eligibility Criteria

Studies were included if they met the following criteria: (1) systematic reviews or meta-analyses of randomized controlled trials (RCTs) or quasi-RCTs focused on acupuncture and moxibustion for PSD; (2) participants diagnosed with PSD, confirmed by computed tomography or magnetic resonance imaging, regardless of gender, age, ethnicity, education, or economic status; (3) interventions applied to the experimental group included acupuncture, moxibustion, electroacupuncture, manual acupuncture, acupoint catgut embedding, or transcutaneous electrical acupoint stimulation, alone or in combination with Western medicine or basic medications. Control groups received interventions other than acupuncture or moxibustion (eg, Western medicine, placebo, and sham acupuncture) to compare the efficacy of acupuncture with other treatment methods or placebo in the treatment of PSD; (4) main outcome indicators included overall effectiveness rate, Hamilton Depression Rating Scale (HAMD), Self-Rating Depression Scale (SDS), and the modified Edinburgh-Scandinavian stroke scale scores; and (5) no restrictions were imposed on the duration of the follow-up period (Table S2 in Multimedia Appendix 1 [16,19-41]).

Exclusion criteria were (1) systematic reviews or meta-analyses that did not include RCTs; (2) studies involving patients without a clear diagnosis of PSD; (3) control group interventions that included acupuncture; (4) duplicate publications; (5) protocols for systematic reviews, commentaries, or conference abstracts; and (6) studies lacking complete or essential information.

### Literature Selection

All studies identified in the search were imported into EndNote (EndNote X20; Clarivate) for deduplication. Two independent reviewers (LM and CLX) screened titles and abstracts according to the inclusion and exclusion criteria and further independently assessed the full text of potentially eligible studies. Disagreements were resolved by discussion, with a third reviewer (XCT) involved when necessary.

### Data Extraction

Data extraction was conducted independently by 2 reviewers (LM and CLX). Extracted data included (1) bibliographic details (title, authors, and year of publication); (2) number and sample size of included studies; (3) intervention methods, control interventions, outcome results, 95% CI, *I*² statistics, *P* values, and study conclusions; and (4) methodological quality assessments of the included studies. Any discrepancies were resolved by discussion with a third reviewer (XCT).

### Quality Assessment

#### Methodological Quality

The included systematic reviews were assessed using the Assessment of Multiple Systematic Reviews-2 (AMSTAR 2) tool [42]. Items 2, 4, 7, 9, 11, 13, and 15 were considered critical domains. Reviews were categorized as “high” quality (no noncompliance on critical items), “moderate” quality (noncompliance on noncritical items only), “low” quality (1 critical item noncompliant), and “very low” quality (more than 1 critical item noncompliant).

In addition, we used the Cochrane Risk-of-Bias tool to reassess the risk of bias in the original RCTs included in the systematic reviews and performed statistical analyses on items with a high risk of bias, such as random sequence generation, allocation concealment, and blinding.

#### Reporting Quality

PRISMA 2020 [18] was used to evaluate reporting quality. Each of the 27 items was scored as 1 (fully reported), 0.5 (partially reported), or 0 (not reported), for a maximum score of 27. Scores <15 indicated “severely deficient” reporting, 15.5‐21 “somewhat deficient,” and 21.5‐27 “relatively complete” [43].

#### Evidence Quality

The Grading of Recommendations Assessment, Development and Evaluation (GRADE) system [44,45] was used to assess the quality of each outcome. Evidence was categorized as high, moderate, low, or very low, in accordance with Cochrane and contemporary guidelines [46]. Quality assessments were carried out independently by 2 reviewers (LM and XXH), with disagreements resolved through discussion or consultation with a third reviewer (XCT).

### Heterogeneity Assessment

Statistical heterogeneity among studies was assessed using the *I*² statistic, quantifying the percentage of variability due to heterogeneity rather than chance [47,48]. *I*² values of 25%, 50%, and 75% were considered low, moderate, and high heterogeneity, respectively [49].

### Data Synthesis and Analysis

A narrative synthesis was performed, focusing on study population, sample size, interventions, outcome indicators, relative effect sizes, *I*² statistics, *P* values, and main conclusions. Where possible, results were also summarized in a tabular form. All collected data were entered into Microsoft Excel 2019 for qualitative synthesis. Overlap of primary studies was assessed using the corrected covered area (CCA) method and calculated as follows: CCA (%)=(N-r)/(rc-r), where N is the total number of included observations from all reviews, r is the number of unique primary studies, and c is the number of systematic reviews. Interpretation was categorized as 0%‐5% (slight overlap), 6%‐10% (moderate overlap), 11%‐15% (high overlap), and >15% (very high overlap).

## Results

### Literature Search Results

**Figure 1.**
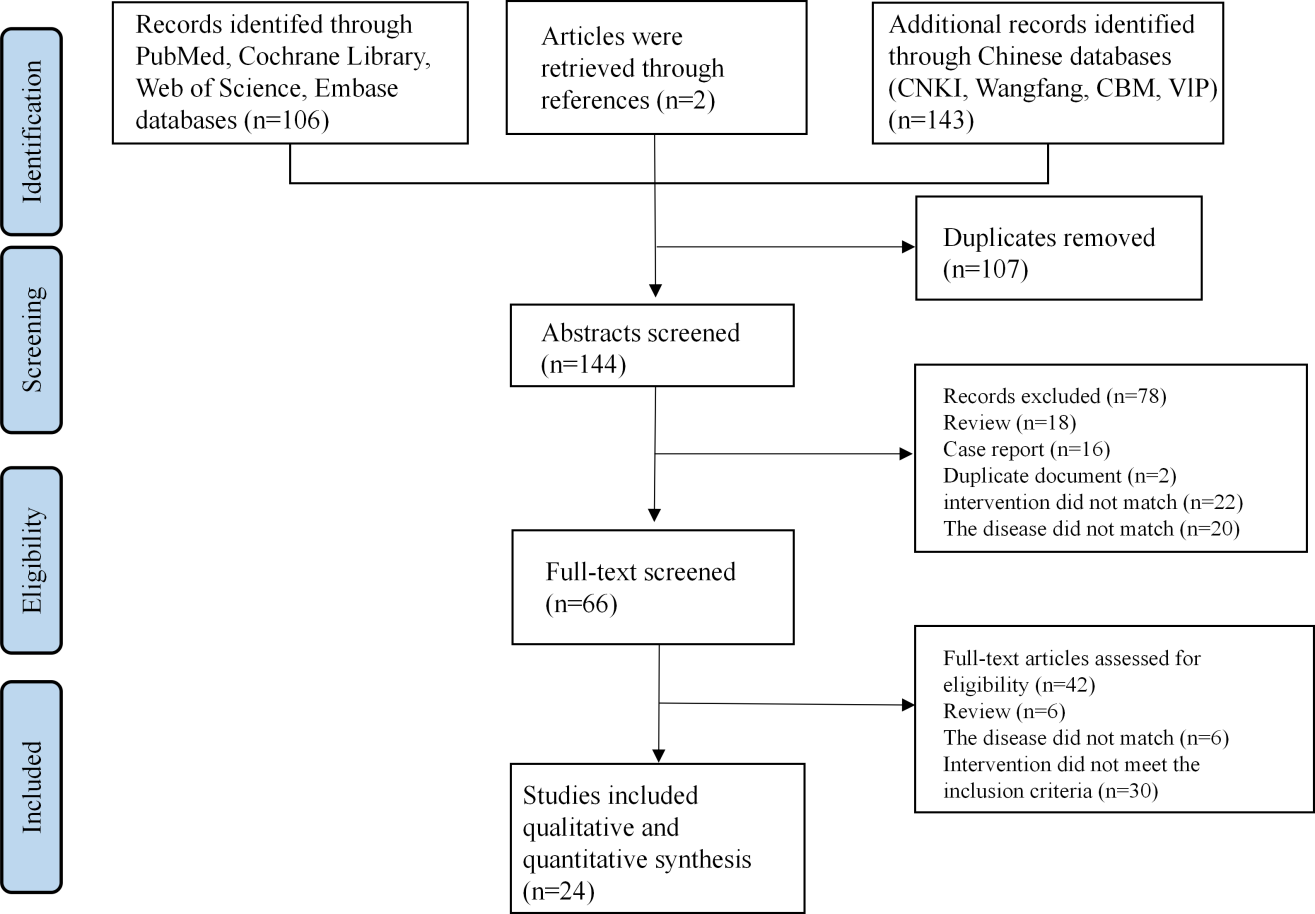
Flow diagram of the search and screening of the studies included in this overview. CBM: Chinese Biomedical Literature Database; CNKI: China National Knowledge Infrastructure.

A total of 251 records were screened for eligibility. After removing 107 duplicates, 144 articles remained. Upon review of titles and abstracts, 78 articles were excluded, with an additional 42 excluded after full-text assessment. Ultimately, 24 articles [16,19-41] met the inclusion criteria for this overview (Figure 1).

### Study Characteristics

The included studies comprised 24 systematic reviews and meta-analyses, with 11 papers published in English and the remaining in Chinese [20,21,24-26,28,29,31,33,37-39,41] (Table 1). Interventions in the experimental group included acupuncture, electroacupuncture, scalp acupuncture, alone or in combination with Western medicine. Comparator groups received Western medicine, sham acupuncture, placebo, or no treatment. All systematic reviews evaluated the methodological quality of their included studies, with 17 [16,20,23-26,28-30,32,34,35,38-41] using the Cochrane Collaboration Risk of Bias tool, 3 [19,21,33] using the Jadad scale, and 3 [27,31,37] combining both tools. A total of 21 systematic reviews [16,19,21,23-26,28-41] concluded that acupuncture was effective for the treatment of PSD, while 3 [20,22,27] reviews found insufficient evidence to support its benefits. Acupuncture was associated with fewer adverse events than antidepressants.

**Table 1.** Baseline characteristics of included reviews.

Study	Trials (subjects)	Experimental intervention	Control intervention	Result summary	Quality assessment
Zhang et al (2010) [19]	14 (1512)	AT^a^	WM^b^/WC^c^	Acupuncture therapy is safe and effective in treating PSD^d^, and could be considered an alternative option for the disorder.	Jadad
Xiong et al (2010) [20]	20 (2031)	AT/EA^e^+WM	WM	Acupuncture is not inferior to Western medicine, and it is worth noting that acupuncture is associated with few adverse reactions.	Cochrane
Lai et al (2012) [21]	29 (2394)	AT/EA+WM	WM	Electroacupuncture combined with fluoxetine improves depression status more significantly than fluoxetine alone in patients with PSD.	Jadad
Zhang et al (2012) [22]	15 (1096)	AT	WM	Comparison between the acupuncture group and Western medicine group in treating PSD revealed that there is a statistical difference in curative rate and remarkably effective rate, but no difference in effective rate.	Grade scale
Zhang et al (2014) [23]	13 (845)	AT/EA/MT^f^+WM	WM	Treating PSD with acupuncture was more effective compared with Western medicine, but the result was less reliable and the quality of evidence was poor.	Cochrane
Tan et al (2016) [24]	14 (1180)	EA	WM	EA may be a more effective and safer treatment for PSD than antidepressants.	Cochrane
Wang et al (2016) [25]	27 (1729)	AT/AT+WM	WM	The therapeutic effect of acupuncture in the treatment of PSD is superior to that of Western medicine	Cochrane
Li et al (2017) [26]	10 (806)	AT+WM	WM	Acupuncture treatment of PSD is more efficient than fluoxetine hydrochloride treatment.	Cochrane
Li et al (2018) [27]	18 (1536)	EA	WM/SAT^g^/UC^h^	There was no significant difference between EA and antidepressants in the severity of depression; however, EA caused fewer adverse events than antidepressants.	Cochrane+ Jadad
Huang et al (2018) [28]	13 (1193)	AT/EA+WM	WM	Acupuncture and electro-acupuncture are effective therapy methods in improving the state of depression of patients with PSD, and they can obviously improve the effect of treatment when combined with Western medicine.	Cochrane
Que et al (2018) [29]	18 (1813)	AT/EA+WM	WM	Compared with Prozac, acupuncture treatment for PSD had fewer adverse effects and fewer symptoms, but there was little difference in clinical efficacy and reduction in HAMD^i^ scores.	Cochrane
Zhang et al (2019) [30]	7 (514)	AT/MT	WM/CHM^j^	Acupuncture is an effective and safe treatment for PSD.	Cochrane
Xu et al (2019) [31]	19 (1542)	AT+WM	SAT/MT	Acupuncture is an effective and safe treatment for PSD. The acupuncture group is more effective than the conventional drug group.	Cochrane+ Jadad
Zhang et al (2020) [32]	13 (904)	AT+WM/EA+WM	WM	Acupuncture combined with antidepressants is an effective treatment for PSD.	Cochrane
Yin et al (2020) [33]	15 (1503)	AT	WM	Acupuncture is more effective than conventional medication in treating PSD.	Jadad
Liu et al (2021) [34]	17 (1402)	AT/EA	WM/SAT/UC	Acupuncture could reduce the degree of PSD.	Cochrane
Zhang et al (2021) [35]	14 (1124)	AT/EA	WM	Acupuncture not only can reduce the severity of PSD, but also has significant effects on decreasing the appearance of other adverse events	Cochrane
Wang et al (2021) [36]	19 (1606)	EA	WM	Compared with antidepressants, electroacupuncture is not less effective at improving depression symptoms in patients with PSD, with greater safety.	Cochrane
Wang et al (2021) [37]	8 (769)	AT/EA(+WM)	WM	Compared with fluoxetine, acupuncture has high clinical efficacy in treating PSD and has significant advantages in improving patients’ symptoms and reducing adverse effects.	Cochrane+ Jadad
Zhang et al (2021) [38]	14 (1120)	AT/MT/AT+MT	WM	Acupuncture and moxibustion treatment are superior to antidepressants in improving the depressive state and activities of daily living of patients with PSD.	Cochrane
Lin et al (2022) [39]	16 (1618)	AT/AT+WM	WM	The Xing nao Kai qiao acupuncture therapy can significantly improve emotional well-being and neurological function in patients with PSD.	Cochrane
Zhong et al (2023) [16] []	11 (1225)	SA+CT^k^	CT	SA^l^ combined with CT can effectively improve the treatment’s success rate for PSD and reduce the severity of depressive symptoms measured by the Self-Rating Depression Scale.	Cochrane
Jiang et al (2023) [40]	14 (1263)	SA/EA+WM	WM	Scalp acupuncture has superior efficacy and safety compared to Western medicine for PSD.	Cochrane
Yang et al (2024) [41]	15 (1016)	EA/EA+WM	WM	Electroacupuncture for PSD can reduce HAMD, NIHSS^m^, and SDS^n^ scores, with significant clinical efficacy and no toxic side effects.	Cochrane

a AT: acupuncture therapy.

b WM: Western medicine.

c WC: waist circumference.

d PSD: poststroke depression.

e EA: electroacupuncture.

f MT: mirror therapy.

g SAT: sham acupuncture therapy.

h UC: ulcerative colitis.

i HAMD: Hamilton Depression Rating Scale.

j CHM: Chinese herbal medicine.

k CT: computed tomography.

l SA: scalp acupuncture.

m NIHSS: National Institutes of Health Stroke Scale.

n SDS: Self-Rating Depression Scale.

### Methodological Appraisal

**Figure 2.**
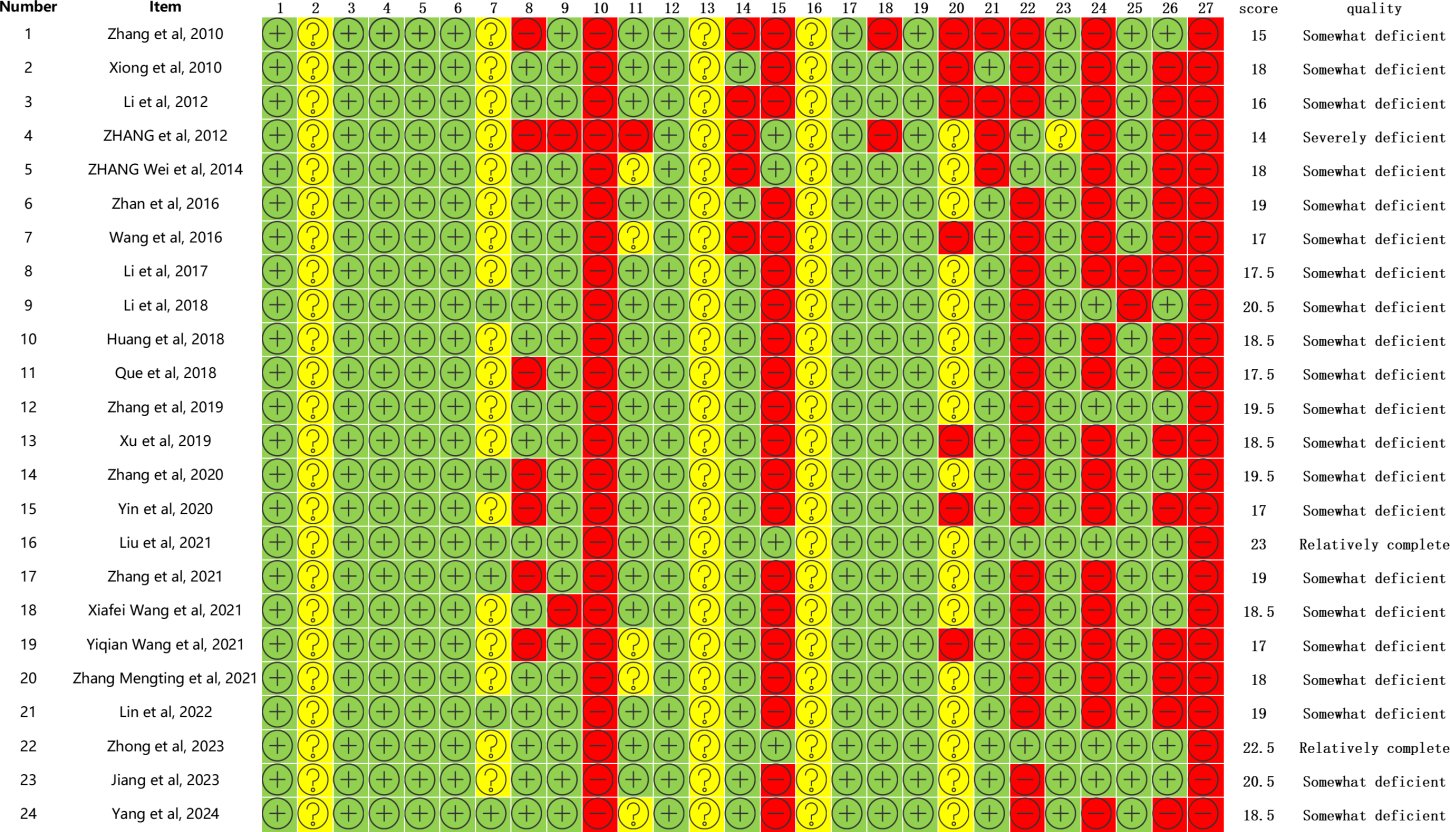
Results of the methodological quality evaluation using Assessment of Multiple Systematic Reviews-2 (AMSTAR 2). “+” denotes yes, “–” denotes no, and “?” denotes partial yes.

The methodological quality of the included reviews was assessed using the AMSTAR 2 tool. Every review had at least 1 critical domain rated as “very low” in methodological quality. In particular, major deficiencies were noted in protocol registration (Item 2), provision of excluded studies (Item 7), and comprehensive assessment of publication bias (Item 15; Figure 2). Out of the 24 included studies, 22 [16,19-26,28-39,41] were rated as “critically low” and 2 [27,40] as “low” in methodological quality. Notably, most studies (21/24) reporting positive findings for acupuncture had critically low methodological quality, while all 3 reviews [20,27,29] indicating insufficient evidence also had methodological shortcomings.

### Reporting Quality Appraisal

**Figure 3.**
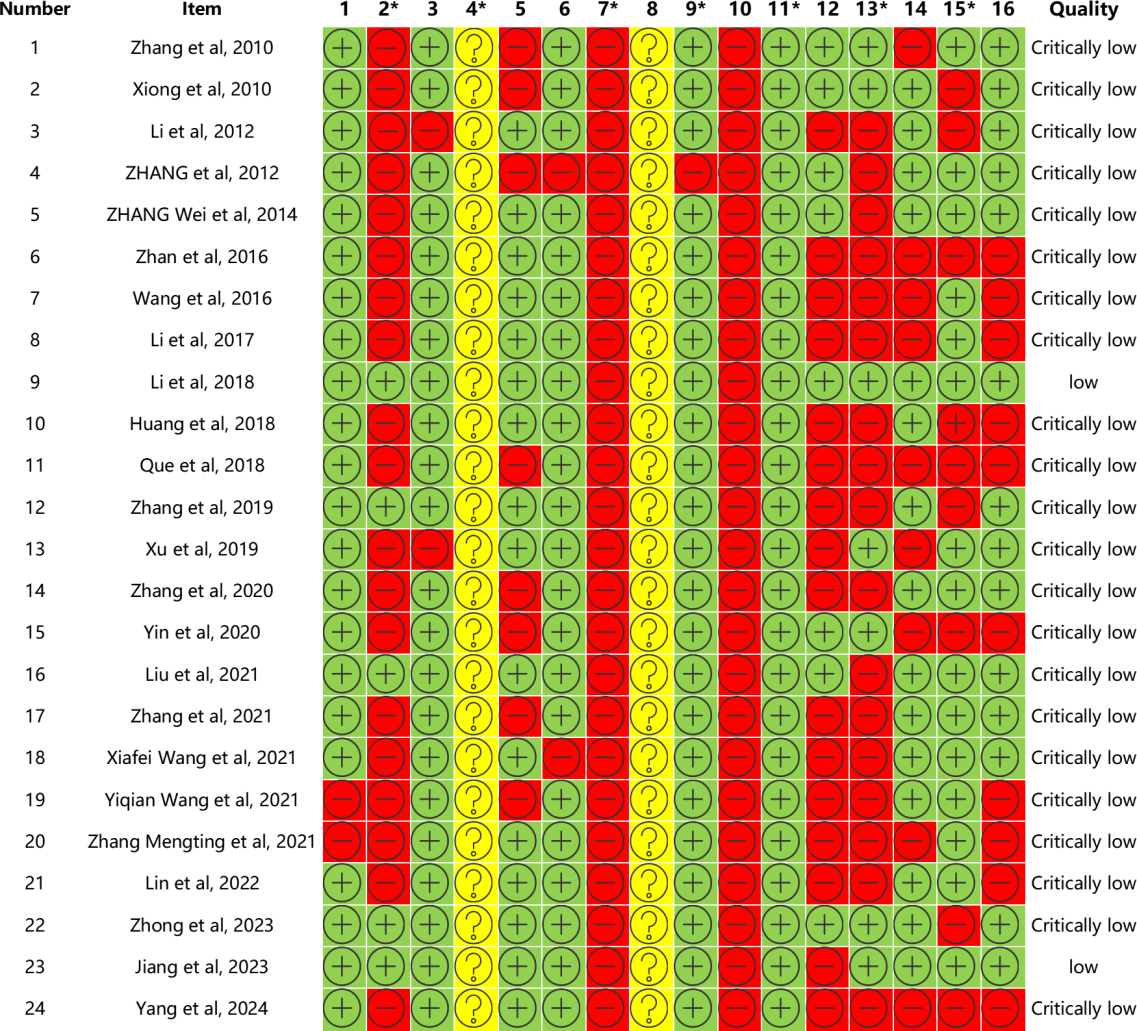
Results of the reporting quality evaluation of Preferred Reporting Items for Systematic reviews and Meta-Analyses (PRISMA). “+” denotes yes, “–” denotes no, and “?” denotes partial yes.

Reporting quality was evaluated using the PRISMA 2020 checklist. Scores for the 24 studies ranged from 14 to 23. One study [22] was classified as “severely deficient” (score <15), 21 studies [19-21,23-33,35-41] as “somewhat deficient” (score 15‐21), and 2 studies [34,40] as “relatively complete” (>21). Underreported items included data items (10), methods for evaluating quality of evidence for each outcome (15), presentation of evidence grading (22), registration and protocol (24), competing interests (26), and public information reporting (27) (Figure 3).

### Risk of Bias in Original Literature

**Figure 4.**
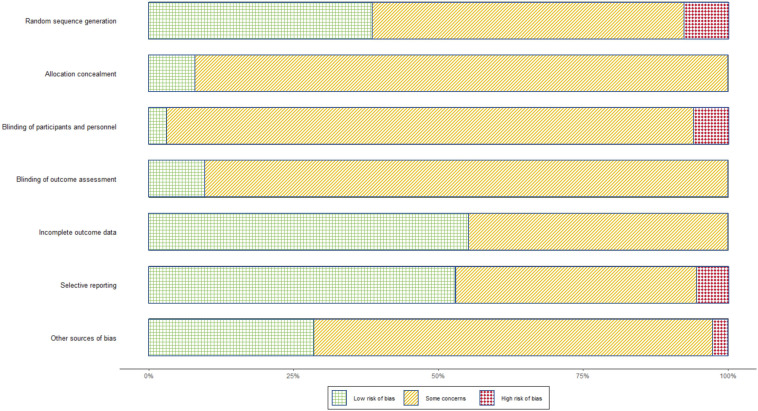
Summary chart of bias risks in original literature. Green squares represent low risk, yellow stripes represent some concerns, and red diamonds represent high risk.

After deduplication, 222 original studies remained. Of these, 46 articles (20.7%) were classified as high risk of bias, while the remainder were labeled as having “some concern.” Among the high-risk domains, random sequence generation constituted 8.6% (19/222), blinding of participants and personnel 7.7% (17/222), selective reporting 4.5% (10/222), and other biases 1.4% (3/222) (Figure 4). Further details can be found in Multimedia Appendix 2. in

### Classification of Evidence Quality

The quality of evidence for the outcome indicators, covering 6 different intervention types, ranged from very low to high. Common downgrading factors included high risk of bias, significant heterogeneity, small sample sizes, and inconsistency of results, as outlined in Table 2 [16,19-41].

**Table 2.** Quality of evidence in included systematic reviews with Grading of Recommendations Assessment, Development, and Evaluation.

Studies and outcomes	Number of outcome studies	Publication bias	Risk of bias	Imprecision	Inconsistency	Indirectness	Quality of evidence
Zhang et al (2010) [19]							
Clinical response	13	−1^a^	−1^b^	0	0	0	Low
Reducing depression severity	14	0	−1^b^	0	−1^c^	0	Low
Xiong et al (2010) [20]							
24 HAMD^d^ score reduction rates	5	0	0	0	0	0	High
24-HAMD	9	0	−1^b^	0	−1^c^	0	Low
SDS^e^	5	0	0	0	−2^f^	0	Low
Li et al (2012) [21]							
HAMD	8	0	−1^b^	0	0	0	Moderate
Zhang et al (2012) [22]							
Curative rate	15	0	−1^b^	0	0	0	Moderate
Effective rate	15	0	−1^b^	0	0	0	Moderate
Zhang et al (2014) [23]							
HAMD	12	0	−1^b^	0	0	−1^g^	Low
Adverse events	8	0	−1^b^	−1^h^	0	0	Low
Tan et al (2016) [24]							
HAMD	12	−1^a^	−1^b^	0	−2^f^	0	Very low
Effective rate	10	−1^a^	−1^b^	0	0	0	Low
Wang et al (2016) [25]							
HAMD	18	−1^a^	0	0	−1^c^	0	Low
BI^i^	4	0	0	−1^h^	−2^f^	0	Very Low
Li et al (2017) [26]							
HAMD	11	0	−1^b^	0	−2^f^	0	Very Low
Curative rate	8	0	−1^b^	0	0	0	Moderate
Effective rate	9	0	−1^b^	0	0	0	Moderate
Adverse events	2	0	0	−1^h^	0	0	Low
Li et al (2018) [27]							
HAMD	15	0	0	0	0	0	High
Huang et al (2018) [28]							
HAMD	13	0	−1^b^	−1^h^	−1^c^	0	Very low
Que et al (2018) [29]							
Effective rate	11	0	−1^b^	0	0	0	Moderate
HAMD-17	5	0	0	−1^h^	−2^f^	0	Very low
HAMD-24	7	0	−1^b^	0	−2^f^	0	Very low
Adverse events	9	0	−1^b^	0	0	0	Moderate
Zhang et al (2019) [30]							
Effective rate	7	0	−1^b^	0	0	0	Moderate
Chen et al (2019) [31]							
HAMD	19	0	0	0	−2^f^	0	Low
BI	4	0	0	−1^h^	−2^f^	0	Very low
Effective rate	12	0	0	0	0	0	High
Adverse events	5	0	0	−1^h^	0	0	Moderate
Zhang et al (2020) [32]							
HAMD	13	−1^a^	−1^b^	0	0	0	Low
Effective rate	6	0	−1^b^	−1^h^	0	0	Low
NIHSS^j^	4	0	−1^b^	−1^h^	−1^c^	0	Very low
BI	3	0	−1^b^	−1^h^	0	0	Low
Xiao et al (2020) [33]							
Effective rate	15	0	−1^b^	0	0	0	Moderate
HAMD	15	0	−1^b^	0	−2^f^	0	Very low
Liu et al (2021) [34]							
HAM-D17	8	0	−1^b^	−1^h^	0	0	Low
HAM-D24	4	0	−1^b^	−1^h^	−1^c^	0	Very low
HAMD	6	0	−1^b^	−1^h^	0	0	Low
Adverse events	7	0	−1^b^	v1^h^	0	-1^g^	Very low
Zhang et al (2021) [35]							
HAMD	12	0	−1^b^	0	−2^f^	0	Very low
NIHSS	2	0	−1^b^	−1^h^	0	0	Low
TESS^k^	3	0	−1^b^	−1^h^	−1^c^	0	Very low
Effective rate	12	−1^a^	−1^b^	0	0	0	Low
Wang et al (2021) [36]							
HAMD	19	0	−1^b^	−1^h^	0	0	Low
Adverse events	9	0	−1^b^	0	0	0	Moderate
Wang et al (2021) [37]							
Effective rate	7	−1^a^	0	0	0	0	Moderate
HAMD	7	0	0	0	−1^c^	0	Moderate
Zhang et al (2021) [38]							
HAM-D24	10	0	−1^b^	0	0	0	Moderate
HAM-D17	3	0	0	−1^h^	−1^c^	0	Low
HAMD score reduction rates	11	0	−1^b^	0	0	0	Moderate
NIHSS	6	0	−1^b^	0	−2^f^	0	Very low
BI	9	0	−1^b^	0	−1^c^	0	Low
Lin et al (2022) [39]							
HAMD	15	0	−1^b^	0	−2^f^	0	Very low
SDS	2	0	0	−1^h^	−1^c^	0	Low
NIHSS	2	0	0	−1^h^	−2^f^	0	Very low
Effective rate	9	0	−1^b^	0	0	0	Moderate
Zhong et al (2023) [16]							
Effective rate	8	0	−1^b^	0	0	0	Moderate
HAMD	7	0	−1^b^	0	−2^f^	0	Very low
SDS	3	0	0	0	−2^f^	0	Low
Jiang et al (2023) [40]							
Effective rate	12	−1^a^	−1^b^	0	0	0	Low
HAMD	10	0	−1^b^	0	−2^f^	0	Very low
Neurological deficit score	6	0	−1^b^	−1^h^	−2^f^	0	Very low
Adverse events	5	0	−1^b^	0	0	0	Moderate
Yang et al (2024) [41]							
HAMD	13	0	−1^b^	0	−2^f^	0	Very low
NIHSS	2	0	0	−1^h^	−2^f^	0	Very low
SDS	B3	0	−1^b^	−1^h^	−2^f^	0	Very low

a Asymmetric funnel plots or all positive results may have a large publication bias.

b Risk of bias in included studies with respect to randomization, blinding, allocation concealment, completeness of outcome data, or selective reporting risk of bias.

c Included in the study 50%≤*I*2<75%.

d HAMD: Hamilton Depression Rating Scale.

e SDS: Self-Rating Depression Scale.

f Included in the study 75%≤*I*2<100%.

g Differences in stroke courses and depression degree exist in the included research.

h Inclusion of too small a sample size for the study (sample size for continuous variables <400).

i BI: Barthel index.

j NIHSS: National Institutes of Health Stroke Scale.

k TESS: Treatment Emergent Symptom Scale.

### Heterogeneity Assessment

Of the reported outcomes, 25 of 60 (42%) demonstrated no heterogeneity (*I*²<25%), 11 of 60 (18%) showed low heterogeneity (25% ≤*I*²<50%), 8 of 60 (13%) moderate heterogeneity (50% ≤*I*²<75%), and 16 of 60 (27%) high heterogeneity (*I*²≥75%). Meta-analyses with high heterogeneity primarily used clinical effective rate and HAMD score as outcomes (Table S2 in Multimedia Appendix 1) [16,19-41].

### Overlap of Primary Studies

The primary research indicators we observed were mostly mild to moderate overlap (Table 3). A CCA <10% may suggest incomplete coverage of original studies in the field, with key clinical trials not being adequately included (eg, publication bias of negative results), and a lack of targeted evidence for subgroup populations (eg, different stroke types and severity of depression).

**Table 3.** Level of overlap for primary studies among the included systematic reviews.

Outcomes	N^a^	r^b^	c^c^	CCA^d^ (%)	Interpretation of overlap
HAMD-24^e^	30	25	4	6.7	Moderate overlap
HAMD-17	15	13	3	7.7	Moderate overlap
HAMD	223	153	18	3	Mild overlap
24 HAMD score reduction rates	17	17	2	0	Mild overlap
Clinical response	13	13	1	0	Mild overlap
Effectiveness rate	121	93	12	2.7	Mild overlap
Curative rate	23	23	2	0	Mild overlap
Barthel index	21	19	3	5.3	Moderate overlap
Treatment Emergent Symptom Scale	3	3	1	0	Mild overlap
SDS^f^	13	12	4	2.8	Mild overlap
NIHSS^g^	17	16	5	1.6	Mild overlap
Barthel index	3	3	1	0	Mild overlap
Adverse events	53	39	8	5.1	Moderate overlap

a N: the total number of included observations from all reviews.

b r: the number of unique primary studies.

c c: the number of systematic reviews.

d CCA: corrected covered area.

e HAMD: Hamilton Depression Rating Scale.

f SDS: ﻿Self-Rating Depression Scale.

g NIHSS: National Institutes of Health Stroke Scale.

## Discussion

### Main Findings

Acupuncture therapy is increasingly used for PSD in clinical practice, primarily due to its capacity to mitigate depressive symptoms, enhance patients’ quality of life, and diminish the adverse side effects typically associated with pharmacological treatments. In recent years, the body of systematic reviews on acupuncture for PSD has grown substantially. This study undertook a comprehensive evaluation of the existing literature, focusing on methodological quality, reporting quality, and evidence quality, to elucidate current weaknesses and provide guidance for future research. Overall, our findings indicate that the included systematic reviews and meta-analyses generally experienced low methodological, reporting, and evidence quality, largely a result of the poor quality of primary studies, especially due to high risks of bias such as inadequate randomization and insufficient allocation concealment. Furthermore, the primary research indices demonstrated mainly mild to moderate overlap, suggesting that coverage of original studies in this field remains incomplete, leaving potentially pivotal clinical trials unincorporated.

### Potential Mechanism

The pathogenesis of PSD is multifactorial. Two major hypotheses involve neurotransmitter imbalance and glutamate-mediated excitotoxicity [50]. The monoaminergic mechanism posits that lower levels of monoamines—such as serotonin, norepinephrine, and dopamine—are associated with depressive symptoms [51]. In addition, glutamate, the brain’s principal excitatory neurotransmitter, is often elevated in patients with PSD, reflecting a state of excitotoxicity and contributing to depressive pathology [52].

Acupuncture is believed to modulate these neurochemical disturbances. Animal and clinical studies suggest that acupuncture elevates levels of serotonin, norepinephrine, and DA in the brain, normalizes glutamate concentrations, and upregulates key neurotransmitter receptors with fewer desensitization issues compared with pharmacotherapy [53-55]. Beyond neurotransmitter effects, acupuncture exerts anti-inflammatory actions, reducing levels of proinflammatory cytokines and improving immunological balance—processes increasingly recognized as pivotal in PSD progression [56-61]. Furthermore, acupuncture appears to modulate the hypothalamic–pituitary–adrenal axis, helping to normalize neuroendocrine function, and may also reduce neuronal apoptosis, particularly within the hippocampus, which is closely linked to mood regulation [62-65].

### Quality Summaries

The methodological quality of the included reviews was evaluated using the AMSTAR 2 system. The methodological strengths of the included reviews were the use of appropriate methods for outcome statistics (24/24, 100%), a satisfactory assessment of risk of bias (24/24, 100%), and study selection and data extraction in duplicate (15/24, 62.5%). Common methodological weaknesses included the failure to provide preregistered protocols (5/24, 20.8%), explain ROB in individual studies when interpreting results (7/24, 29.2%), provide a full list of excluded studies (0/24, 0%), and extract data on funding sources (0/24, 0%).

The methodological quality of reviews was systematically assessed using AMSTAR 2. All included reviews applied appropriate statistical methods and risk of bias assessments (24/24, 100%) and most performed duplicate selection and data extraction (15/24, 62.5%). However, major shortcomings were evident: only a minority preregistered protocols (5/24, 20.8%), few explained the risk of bias in interpreting results (7/24, 29.2%), none provided a list of excluded studies (0/24), and none extracted funding source data (0/24). The absence of protocol registration and transparency in study selection raises concerns about selective reporting.

According to the GRADE system, although acupuncture appeared more effective than controls for PSD, none of the 60 outcome variables achieved a high-quality evidence rating, and only 8 were rated as moderate quality. Most underlying RCTs were small, single-center studies, with risk of bias, mostly from inadequate blinding, being the most prominent factor for downgrading.

Regarding PRISMA 2020 criteria, the reporting quality was generally suboptimal: only 1 review [22] had major defects, 21 had certain deficiencies, and 2 [16,34] met standards for completeness. Notably, most reviews neglected protocol registration, subgroup or sensitivity analyses for heterogeneity, or evidence grading for outcomes. Several lacked key visualizations such as flow or bias charts, and some failed to report funding or conflicts of interest, compromising the transparency and objectivity of their results.

### Outlook and Recommendations

Almost all of the included SRs exhibited low or very low methodological quality, indicating an urgent need to improve the conduct and reporting of these reviews. Acupuncture shows promise as a complementary approach for improving depressive symptoms after stroke. However, these findings highlight a pressing need for large, well-designed, randomized controlled trials and higher-quality systematic reviews. Assessments typically rely on scales such as HAMD, SDS, and the modified Edinburgh-Scandinavian stroke scale, but these are somewhat subjective and may introduce bias. Future research should emphasize blinding (of both patients and outcome assessors), clearer methodology, robust protocol registration, and transparency regarding funding and conflicts of interest, in order to improve the reliability and applicability of the evidence. In addition, the results showed that there was mainly mild to moderate overlap, and key clinical trials may not have been included. Follow-up studies need to increase the coverage of clinical trials and include multiple languages and countries.

### Strengths and Limitations

A principal strength of this review lies in its focus on meta-analyses of RCTs, representing the highest tier of evidence, with rigorous eligibility criteria to minimize confounding. Nonetheless, several limitations should be acknowledged: (1) interventions were heterogeneous across studies, precluding quantitative synthesis; (2) the included systematic reviews generally exhibited low methodological, reporting, and evidence quality; (3) the lack of differentiation between different subtypes of PSD and TCM syndromes complicates the interpretation of the evidence for acupuncture in treating PSD; and (4) the subjective nature of AMSTAR 2 and PRISMA assessments could introduce rater bias.

### Conclusions

Overall, the preponderance of current systematic reviews suggests a positive effect of acupuncture in the management of PSD. However, the evidence base is undermined by pervasive deficiencies in methodological rigor and quality of reporting. To advance the field and inform clinical decision-making, further high-quality multicenter RCTs and rigorously conducted systematic reviews are imperative.

## Supplementary material

10.2196/76577 Multimedia Appendix 1 Detailed search strategies for each database and main outcomes and evidence quality.

10.2196/76577 Multimedia Appendix 2 Full details of references.

10.2196/76577 Checklist 1 PRISMA checklist.

## Abbreviations

AMSTAR 2: Assessment of Multiple Systematic Reviews-2
CCA: corrected covered area
GRADE: Grading of Recommendations Assessment, Development and Evaluation
HAMD: Hamilton Depression Rating Scale
PRISMA: Preferred Reporting Items for Systematic Reviews and Meta-Analyses
PSD: poststroke depression
RCT: randomized controlled trial
SDS: Self-Rating Depression Scale
