# Supplementary material for: Acupuncture and Moxibustion for Poststroke Depression: Systematic Review
Source: Interact J Med Res. 2025 Oct 16;14:e76577. doi: 10.2196/76577 (PMC12530694; doi:10.2196/76577)
Supplement: Multimedia Appendix 1 [file ijmr-v14-e76577-s001.docx]

**Table Sl.** Search strategies.

| Database |  |
| --- | --- |
| CNKI | SU%=(‘针灸’ + ‘针刺' + ‘针' + ‘艾灸' + ‘灸' + ‘手针' + ‘穴位埋线’ + ‘经皮穴位电刺激')*(‘系统评价' + ‘系统综述' + ‘荟萃分析' + ‘Meta 分析' + ‘循证评价')*(‘卒中后抑郁' + ‘中风后抑郁' + ‘PDS') |
| Wanfang | 主题:((“针灸” or “针刺” or“针”or“艾灸”or“灸” or “手针” or “穴位埋线'” or “经皮穴位电刺激”) and (“系统评价”or“系统综述”or“荟萃分析”or “Meta 分析” or “循证评价”) and (“卒中后抑郁” or “中风后抑郁” or “PDS”)) |
| VIP | M=((“针灸” or “针刺” or“针” or “艾灸”or“灸” or “手针” or “穴位埋线'” or “经皮穴位电刺激”) and (“系统评价” or “系统综述” or “荟萃分析”or “Meta 分析” or “循证评价”) and (“卒中后抑郁” or “中风后抑郁” or “PDS”)) |
| CBM | ((“针灸” or “针刺” or“针”or“艾灸”or“灸” or “手针” or “穴位埋线'” or “经皮穴位电刺激”) and (“系统评价” or “系统综述” or “荟萃分析”or “Meta 分析” or “循证评价”) and (“卒中后抑郁” or “中风后抑郁” or “PDS”)) |
| PubMed | ((acupuncture OR electroacupuncture OR moxibustion OR “manual acupuncture” OR “acupoint catgut embedding” OR “transcutaneous electrical acupoint stimulation” OR “Acupuncture" [Mesh] OR “Electroacupuncture"[Mesh] OR “Moxibustion"[Mesh]) AND (systematic review OR meta-analysis OR “Systematic Review” [Publication Type] OR “Systematic Reviews as Topic"[Mesh] OR “Meta-Analysis as Topic"[Mesh])) AND (“Post-stroke depression” OR “PSD” [Mesh]) |
| Embase | ('acupuncture': ab,ti OR 'electroacupuncture':ab,ti OR 'moxibustion': ab.ti OR ' manual acupuncture': ab,ti OR 'acupoint catgut embedding':ab,ti OR 'transcutaneous electrical acupoint stimulation':ab.ti) AND ('systematic review': ab,ti OR 'meta-analysis': ab,ti)AND ('Post-stroke depression': ab,ti OR 'PSD': ab,ti) |
| CochraneLibrary | #1 (acupuncture OR electroacupuncture OR moxibustion OR “manual acupuncture” OR “acupoint catgut embedding” OR “transcutaneous electrical acupoint stimulation”): ti,ab,kw  #2 (“Post-stroke depression " OR “PDS”): ti.ab.kw  #3(systematic review OR meta-analysis): ti,ab,kw  #1 and #2 and #3 |
| Web of Science | TS= ((acupuncture OR electroacupuncture OR moxibustion OR ‘manual acupuncture’ OR ‘acupoint catgut embedding’ OR ‘transcutaneous electrical acupoint stimulation’) AND (‘systematic review’ OR ‘meta-analysis’) AND (‘Post-stroke depression’ OR ‘PDS’ )) |

CBM: China Biology Medicine; CNKI: China National Knowledge Infrastructure; Embase: Excerpta Medica Database; PDS: Post-stroke depression; VIP:VIP Database for Chinese Technical Periodicals.

| **Table S2.** Main outcomes and evidence quality. | | | | | | | | | | | |
| --- | --- | --- | --- | --- | --- | --- | --- | --- | --- | --- | --- |
| Study (year) | Outcome | Trials (Experiment/Control) | Limitation | Imprecision | Inconsistency | Indirectness | Reporting bias | Quality | Relative effect (95% CI) | I^2^(P) | P |
| Zhang et al (2010) [19] | clinical response | 13(864/708) | -1^a^ | 0 | 0 | 0 | 0 | moderate | RR:1.36(1.24,1.50) | 36%  P =0.10 | ＜0.00001 |
|  | HAMD score | 14(824/688) | -1^a^ | 0 | -1^c^ | 0 | 0 | low | WMD:2.54(1.11,3.97) | 79%  P＜0.00001 | 0.0005 |
| Xiong et al (2010) [20] | 24-item  HAMD reduction rate | 8(271/231) | -1^a^ | 0 | 0 | 0 | -1^e^ | low | RR:1.15(1.07,1.24) | 24%  P =0.26 | ＜0.0001 |
|  | HAMD score | 7(230/228) | -1^a^ | 0 | -1^c^ | 0 | -1^e^ | very low | WMD : -1.34(-2.67,-0.02) | 69%  P=0.003 | 0.05 |
|  | SDS score | 3(98/96) | -1^a^ | -1^b^ | -1^c^ | 0 | -1^e^ | very low | WMD: –6.02( –8.73, –3.30) | 90%  P ＜0.0001 | ＜0.0001 |
| Lai et al (2012) [21] | HAMD score | 8(266/265) | -1^a^ | 0 | 0 | 0 | -1^e^ | low | WMD:-2.20(-2.62,-1.79) | 26%  P=0.22 | ＜0.00001 |
| Zhang et al (2012) [22] | curative rate(HAMD) | 15(553/543) | -1^a^ | 0 | 0 | 0 | -1^e^ | low | OR:1.48(1.10,1.97) | 0%  P=1.00 | 0.008 |
| Zhang et al (2014) [23] | HAMD score | 12(417/374) | -1^a^ | 0 | 0 | 0 | 0 | moderate | SMD :0.26(0.11,0.40) | 29% P=0.16 | =0.0004 |
|  | adverse event | 8(251/251) | -1^a^ | 0 | 0 | 0 | -1^e^ | low | RR:0.32(0.19,0.53) | 0%  P=0.86 | ＜0.00001 |
| Tan et al (2016) [24] | HAMD score | 12(525/515) | -1^a^ | 0 | -1^c^ | 0 | -1^e^ | very low | MD:-0.77(-1.47,-0.07) | 85%  P＜0.00001 | 0.03 |
|  | clinical effective rate | 10(426/416) | -1^a^ | 0 | 0 | 0 | -1^e^ | low | RR:1.11(1.05,1.19) | 0% P=0.65 | 0.0007 |
| Wang et al (2016) [25] | HAMD score | 18(539/538) | -1^a^ | 0 | -1^c^ | 0 | -1^e^ | very low | MD:0.86(0.27,1.45) | 61%  P=0.0004 | 0.004 |
| Li et al (2017) [26] | HAMD score | 11(472/467) | -1^a^ | 0 | 0 | 0 | 0 | moderate | MD:-3.89(-5.43,-2.34) | 0%  P=0.58 | ＜0.00001 |
|  | curative rate | 8(367/366) | -1^a^ | 0 | 0 | 0 | -1^e^ | low | OR:1.84(1.29,2.62) | 0% P=0.93 | 0.0007 |
|  | clinical effective rate | 9(390/386） | -1^a^ | 0 | -1^c^ | 0 | -1^e^ | very low | OR:3.38(1.81,6.29) | 79.2% P=0.03 | 0.0001 |
|  | adverse events | 2(63/60) | 0 | -1^b^ | -1^c^ | 0 | -1^e^ | very low | RR:0.59 (0.20 1.78) | 0% P=0.59 | 0.35 |
| Li et al (2018) [27] | HAMD score | 15(636/595) | -1^a^ | 0 | 0 | 0 | 0 | moderate | SMD:-0.04 (-0.18,0.10) | 28%  P=0.15 | 0.56 |
|  | adverse events | 8(397/401) | -1^a^ | 0 | 0 | 0 | -1^e^ | low | RR:0.21(0.14,0.33) | 0%  P=0.77 | ＜0.00001 |
| Huang et al (2018) [28] | HAMD score(AT/WM) | 3(152/196) | -1^a^ | 0 | -1^c^ | 0 | -1^e^ | very low | MD:-0.54(-1.46,0.39) | 47% P=0.15 | 0.26 |
|  | HAMD score(EA/WM) | 5(200/197) | -1^a^ | 0 | -1^c^ | 0 | -1^e^ | very low | MD:0.13(-1.07,1.33) | 67%  P=0.02 | 0.83 |
|  | HAMD score  (AT+WM/WM) | 3(98/96) | -1^a^ | -1^b^ | 0 | 0 | -1^e^ | very low | MD:-3.28(-4.45,-2.12) | 42%  P=0.18 | ＜0.00001 |
| Que et al (2018) [29] | clinical effective rate(AT/WM) | 11(657/696) | -1^a^ | 0 | 0 | 0 | -1^e^ | low | OR:2.30 (1.66,3.19) | 0%  P=0.62 | ＜0.00001 |
|  | clinical effective rate(EA/WM) | 4(133/131) | -1^a^ | -1^b^ | 0 | 0 | -1^e^ | very low | OR:1.32(0.65,2.68) | 0%  P=1.00 | 0.44 |
|  | HAMD score(AT/WM) | 7(462/457) | -1^a^ | 0 | -1^c^ | 0 | -1^e^ | very low | MD:-2.58(-4.06,-1.09) | 90%  P＜0.00001 | 0.0007 |
|  | adverse events(AT/WM) | 5(315/313) | -1^a^ | 0 | 0 | 0 | -1^e^ | low | OR:0.10(0.05,0.19) | 42%  P=0.09 | ＜0.00001 |
| Zhang et al (2019) [30] | clinical effective rate | 7(260/254) | -1^a^ | 0 | 0 | 0 | -1^e^ | low | RR:1.16(1.08,1.24) | 4%  P=0.40 | ＜0.0001 |
| Xu et al (2019) [31] | HAMD score | 7(462/457) | -1^a^ | 0 | -1^c^ | 0 | -1^e^ | very low | WMD:-4.52(-5.25,-3.79) | 82.4%  P=0.000 | ＜0.0001 |
|  | clinical effective rate | 5(315/313) | -1^a^ | 0 | 0 | 0 | 0 | moderate | RR:1.13(1.00,1.26) | 0%  P=1.000 | ＜0.0001 |
|  | adverse events | 7(260/254) | -1^a^ | 0 | 0 | 0 | -1^e^ | low | RR:0.23(0.11,0.48) | 0%  P=0.681 | ＜0.0001 |
| Zhang et al (2020) [32] | HAMD score | 13(456/448) | -1^a^ | 0 | 0 | 0 | 0 | moderate | WMD:-3.60(-4.25,-2.95) | 43%  P=0.05 | ＜0.0001 |
|  | clinical effective rate | 6(186/183) | -1^a^ | 0 | 0 | 0 | -1^e^ | low | RR:1.33(1.19,1.49) | 0%  P=0.60 | ＜0.0001 |
| Yin et al (2020) [33] | HAMD score | 15(755/748) | -1^a^ | 0 | -1^c^ | 0 | -1^e^ | very low | WMD:-2.98(-3.32,-2.63) | 81%  P＜0.0001 | ＜0.0001 |
|  | clinical effective rate | 15(755/748) | -1^a^ | 0 | 0 | 0 | -1^e^ | low | RR:3.18(2.31,4.37) | 0%  P=0.97 | ＜0.0001 |
| Liu et al (2021) [34] | HAM-D17 scale  (conventional treatment) | 3(126/126) | -1^a^ | -1^b^ | 0 | 0 | -1^e^ | very low | MD:-5.08(-6.48,-3.67) | 0%  P=0.67 | ＜0.0001 |
|  | HAM-D17 scale  (antidepressants) | 6(186/183) | -1^a^ | 0 | -1^c^ | 0 | -1^e^ | very low | MD:-0.43(-1.61,0.75) | 51%  P=0.09 | 0.47 |
|  | HAM-D24 scale  (conventional treatment) | 2(63/60) | -1^a^ | -1^b^ | -1^c^ | 0 | -1^e^ | very low | MD:-9.72(-14.54,-4.91) | 65%  P=0.09 | ＜0.0001 |
|  | HAM-D24 scale  (antidepressants) | 2(60/60) | -1^a^ | -1^b^ | -1^c^ | 0 | -1^e^ | very low | MD:-3.09(-10.81,4.63) | 90%  P=0.001 | 0.43 |
|  | HAM-D  (conventional treatment) | 2(79/78) | -1^a^ | -1^b^ | -1^c^ | 0 | -1^e^ | very low | MD:-2.72(-3.61,-1.82) | 0%  P=0.73 | ＜0.0001 |
|  | HAM-D  (antidepressants) | 4(225/269) | -1^a^ | 0 | -1^c^ | 0 | -1^e^ | very low | MD:-1.55(-4.36,1.26) | 95%  P＜0.00001 | 0.28 |
|  | adverse events | 7(260/254) | -1^a^ | 0 | 0 | 0 | -1^e^ | low | RR:0.63(0.21,1.83) | 38%  P=0.20 | 0.39 |
|  | adverse events | 7(260/254) | -1^a^ | 0 | 0 | 0 | -1^e^ | low | RR:0.16(0.07,0.39) | 35%  P=0.19 | ＜0.0001 |
| Zhang et al (2021) [35] | HAMD score(w6) | 6(205/201) | -1^a^ | 0 | -1^c^ | 0 | -1^e^ | very low | MD:-1.20(-1.92,-0.49) | 93%  P＜0.00001 | 0.0009 |
|  | HAMD score(w8) | 4(224/224) | -1^a^ | 0 | 0 | 0 | -1^e^ | low | MD:-4.33(-4.96,-3.70) | 0%  P=0.58 | ＜0.0001 |
|  | clinical effective rate | 12(507/503) | -1^a^ | 0 | 0 | 0 | 0 | moderate | RR:1.15(1.08,1.21) | 24%  P=0.21 | ＜0.00001 |
| Wang et al (2021) [36] | HAMD score | 22(807/765) | -1^a^ | 0 | -1^c^ | 0 | 0 | low | SMD:-0.15(-0.33,0.03) | 70%  P＜0.00001 | 0.10 |
|  | adverse events | 9(428/431) | -1^a^ | 0 | 0 | 0 | -1^e^ | low | RR:0.21(0.14,0.32) | 0%  P=0.83 | ＜0.00001 |
| Wang et al (2021) [37] | clinical effective rate | 7(339/340) | -1^a^ | 0 | 0 | 0 | -1^e^ | low | RR:1.08(1.01,1.16) | 0%  P=0.80 | 0.02 |
|  | HAMD score | 7(292/268) | -1^a^ | 0 | 0 | 0 | -1^e^ | low | MD:-1.15(-1.97,-0.32) | 55%  P=0.04 | 0.006 |
| Zhang et al (2021) [38] | HAM-D24 scale | 11(356/349) | -1^a^ | 0 | 0 | 0 | -1^e^ | low | MD:-0.58(-0.74,-0.43) | 36%  P=0.12 | ＜0.0001 |
|  | HAM-D17 scale | 3(108/107) | -1^a^ | -1^b^ | -1^c^ | 0 | -1^e^ | very low | MD:-0.76(-1.18,-0.33) | 57%  P=0.10 | 0.0005 |
|  | HAMD reduction rate | 12(469/462) | -1^a^ | 0 | 0 | 0 | 0 | moderate | MD:1.15(1.09,1.21) | 0%  P=0.91 | ＜0.00001 |
| Lin et al (2022) [39] | HAMD score | 15(831/671) | -1^a^ | 0 | -1^c^ | 0 | 0 | low | MD:-4.46(-6.26,-2.67) | 96%  P＜0.00001 | ＜0.00001 |
|  | SDS | 2(238/134) | -1^a^ | -1^b^ | -1^c^ | 0 | -1^e^ | Very low | MD:-7.49(-9.73,-5.26) | 66%  P=0.09 | ＜0.00001 |
|  | clinical effective rate | 9(366/311) | -1^a^ | 0 | 0 | 0 | -1^e^ | low | RR:1.16(1.08,1.26) | 0%  P=0.67 | 0.0001 |
| Zhong et al (2023) [16] | HAMD score | 7 | -1^a^ | 0 | -1^c^ | 0 | -1^e^ | Very low | MD:−3.79(− 8.51, 0.92) | 99%  P＜0.00001 | 0.11 |
|  | SDS | 3 | -1^a^ | -1^b^ | 0 | 0 | -1^e^ | Very low | MD:−8.72(− 9.71, − 7.73) | 81%  P=0.005 | ＜0.00001 |
|  | adverse events | 8 | -1^a^ | 0 | 0 | 0 | -1^e^ | low | OR:2.44(1.61, 3.70) | 0%  P=0.63 | ＜0.0001 |
| Jiang et al (2023) [40] | clinical effective rate | 12(553/543) | -1^a^ | 0 | 0 | 0 | -1^e^ | low | RR:1.09(1.02,1.16) | 11%  P=0.34 | 0.008 |
|  | HAMD score | 10(351/343) | -1^a^ | 0 | -1^c^ | 0 | -1^e^ | very low | MD:−2.29(−3.88, -0.70) | 85%  P＜0.00001 | 0.005 |
|  | adverse events | 5(270/266) | -1^a^ | 0 | 0 | 0 | -1^e^ | low | RR:0.12(0.05,0.29) | 0%  P=0.59 | ＜0.00001 |
| Yang et al (2024) [41] | SDS | 5(186/178) | -1^a^ | -1^b^ | -1^c^ | 0 | -1^e^ | very low | MD:−8.08(−15.45, − 0.72) | 99%  P＜0.00001 | 0.03 |
|  | HAMD score | 13(440/427) | -1^a^ | 0 | -1^c^ | 0 | -1^e^ | very low | MD:-1.28(-1.86,-0.69) | 93%  ＜0.00001 | ＜0.0001 |
| Note: TER: total effective rate WM: western medicine a: Potential bias from randomization methods, allocation concealment, blinding, or selective reporting. b: 95% CI was not narrow enough, or across the null line, or insufficient sample size. c: Small overlap of 95% CIs, inconsistent effects across included trials, or large heterogeneity. d: Different interventions, or inconsistent outcomes. e: funnel diagram shows asymmetry, gray literature was not retrieved and the search database is incomplete; NA: not available; OR: odds ratio; RR: risk ratio; SMD: standard mean difference | | | | | | | | | | | |

**References**

16. Zhong D, Cheng H, Pan Z, et al. Efficacy of scalp acupuncture combined with conventional therapy in the intervention of post-stroke depression: a systematic review and meta-analysis. Complement Ther Med. Oct 2023;77:102975. [doi: ] [Medline: 37619716]

19. Zhang ZJ, Chen HY, Yip K chee, Ng R, Wong VT. The effectiveness and safety of acupuncture therapy in depressive disorders: systematic review and meta-analysis. J Affect Disord. Jul 2010;124(1-2):9-21. [doi: ] [Medline: 19632725]

20. Xiong J, DuYH, Liu JL, et al. Acupuncture versus western medicine for post stroke depression: a systematic review [Article in Chinese]. J Evid Based Med. 2010;10(3):179-185. [doi: ]

21. Lai SW, Chen J, Hu J, Wang H, Wang S. Meta-analysis comparing the effects of combined acupuncture and medicine with Western medicine on improving depressive status in patients with post-stroke depression [Article in Chinese]. Shaanxi Traditional Chinese Medicine. 2012;33(9):1263-1267. URL: <http://dianda.cqvip.com/Qikan/Article/Detail?id=43080267&from=Qikan_Article_Detail> [Accessed 2025-09-19]

22. Zhang GC, Fu WB, Xu NG, et al. Meta analysis of the curative effect of acupuncture on post-stroke depression. J Tradit Chin Med. Mar 2012;32(1):6-11. [doi: ] [Medline: 22594095]

23. Zhang W, Sun J hua, Gao Y, et al. System review on treating post-stroke depression with acupuncture. World J Acupunct Moxibustion. Jun 2014;24(2):52-59. [doi: ]

24. Tan F, Cheng NF, Tan JQ. Systematic evaluation of the efficacy of electroacupuncture and antidepressant western medicine in treating post-stroke depression [Article in Chinese]. J Tradit Chin Med. 2016;34(10):2379-2383. URL: <https://tcmjc.com/zyyxk/articleIndex/crossref?doi=10.13193%2Fj.issn.1673-7717.2016.10.021&utm_source=chatgpt.com> [Accessed 2025-09-12]

25. Wang Q, Zheng S, Tang M, Cui H. Meta-analysis of body acupuncture treatment for post-stroke depression [Article in Chinese]. Journal of Traditional Chinese Medicine. 2016;31(8):1227-1231. URL: <http://dianda.cqvip.com/Qikan/Article/Detail?id=670047378&from=Qikan_Article_Detail> [Accessed 2025-09-19]

26. Li W, Ding SN, Zhang JG. Systematic evaluation of the effects of two methods of treating depressed patients. Journal of Huzhou Normal. 2017;39(10):84-91. URL: <https://www.fx361.com/page/2017/1216/18538576.shtml?utm_source=chatgpt.com> [Accessed 2025-09-12]

27. Li XB, Wang J, Xu AD, et al. Clinical effects and safety of electroacupuncture for the treatment of post-stroke depression: a systematic review and meta-analysis of randomised controlled trials. Acupunct Med. Oct 2018;36(5):284-293. [doi: ] [Medline: 29776950]

28. Huang YJ, Li LX, Zhou YL, Wu J, Lin GH, Ming KW. Meta-analysis of the efficacy of acupuncture and electroacupuncture for post-stroke depression. Mod Hosp. 2018;18(1):120-124. URL: <https://qikan.cqvip.com/Qikan/Article/Detail?id=7111635010> [Accessed 2025-09-12]

29. Que F, Wen Y, Wang W. A systematic review of the efficacy of acupuncture compared with Prozac in the treatment of post-stroke depression [Article in Chinese]. Hunan Journal of Traditional Chinese Medicine. 2018;34(9):141-145. URL: <https://www.hunanzyzz.com/ch/reader/view_abstract.aspx?file_no=201809066&flag=1> [Accessed 2025-09-19]

30. Zhang XY, Li YX, Liu DL, Zhang BY, Chen DM. The effectiveness of acupuncture therapy in patients with post-stroke depression: an updated meta-analysis of randomized controlled trials. Medicine (Baltimore). May 2019;98(22):e15894. [doi: ] [Medline: 31145349]

31. Xu R, Chen J. Meta-analysis and trial sequential analysis of acupuncture treatment for depression in patients with post-stroke depression [Article in Chinese]. Chin J Gerontol. 2019;39(23):5722-5726. URL: <http://dianda.cqvip.com/Qikan/Article/Detail?id=7100428755&from=Qikan_Article_Detail> [Accessed 2025-09-19]

32. Zhang K, Cui G, Gao Y, Shen W. Does acupuncture combined with antidepressants have a better therapeutic effect on post-stroke depression? A systematic review and meta-analysis. Acupunct Med. Oct 2021;39(5):432-440. [doi: ] [Medline: 33334118]

33. Yin M, Wei X, Liang F. Meta-analysis of acupuncture treatment for post-stroke depression [Article in Chinese]. Henan Traditional Chinese Medicine. 2020;40(11):1729-1732. URL: <http://dianda.cqvip.com/Qikan/Article/Detail?id=7103220306&from=Qikan_Article_Detail> [Accessed 2025-09-19]

34. Liu R, Zhang K, Tong QY, Cui GW, Ma W, Shen WD. Acupuncture for post-stroke depression: a systematic review and meta-analysis. BMC Complement Med Ther. Apr 1, 2021;21(1):109. [doi: ] [Medline: 33794857]

35. Zhang L, Chen B, Yao Q, et al. Comparison between acupuncture and antidepressant therapy for the treatment of poststroke depression. Medicine (Abingdon). 2021;100(22):e25950. [doi: ] [Medline: 34087835]

36. Wang X, Cai W, Wang Y, Huang S, Zhang Q, Wang F. Is electroacupuncture an effective and safe treatment for poststroke depression? An updated systematic review and meta-analysis. Biomed Res Int. 2021;2021(1):8661162. [doi: ] [Medline: 34485527]

37. Wang Y, Yuan C, Yang J, Li J, Huang H. A systematic review of the efficacy and safety of acupuncture in treating post-stroke depression [Article in Chinese]. Shandong Journal of Traditional Chinese Medicine. 2021;40(5):464-470. URL: <https://d.wanfangdata.com.cn/periodical/sdzyzz202105004> [Accessed 2025-09-19]

38. Zhang MT, Tan K, Hu S, Liao LY, Shi WY, Zhou WJ. Meta-analysis of acupuncture to improve the depressive state and daily life ability of post-stroke depressed patients [Article in Chinese]. Shandong J Tradit Chin Med. 2021;55(1):13-19. [Medline: 34392647]

39. Lin S, Lu C, Zhuo Y, Yuan J, Zhu Y, Pi M. Meta-analysis of the efficacy and safety of brain-awakening acupuncture in the treatment of post-stroke depression [Article in Chinese]. Journal of Guangzhou University of Chinese Medicine. 2022;39(6):1453-1460. URL: <http://dianda.cqvip.com/Qikan/Article/Detail?id=00002FGN4J4O7JP0MLDO3JP067R&from=Qikan_Article_Detail> [Accessed 2025-09-19]

40. Jiang W, Jiang X, Yu T, Gao Y, Sun Y. Efficacy and safety of scalp acupuncture for poststroke depression: a meta-analysis and systematic review. Medicine (Baltimore). Aug 4, 2023;102(31):e34561. [doi: ] [Medline: 37543780]

41. Yang Y, Cheng Z, Xu W, Ye W, Xiong J, Hong E. Meta-analysis of the effectiveness and safety of electroacupuncture in treating post-stroke depression [Article in Chinese]. Modern Distance Education of Traditional Chinese Medicine. 2024;22(8). URL: <https://qikan.cqvip.com/Qikan/Article/Detail?id=7111635010> [Accessed 2025-09-19]
